# Association Between Okinawan Vegetables Consumption and Risk of Type 2 Diabetes in Japanese Communities: The JPHC Study

**DOI:** 10.2188/jea.JE20180262

**Published:** 2020-05-05

**Authors:** Junpei Yamamoto, Junko Ishihara, Ayaka Kotemori, Takahiro Yoshizaki, Atsushi Goto, Tetsuya Mizoue, Mitsuhiko Noda, Norie Sawada, Shoichiro Tsugane

**Affiliations:** 1 School of Life and Environmental Science, Department of Food and Life Science, Azabu University, Kanagawa, Japan; 2 Epidemiology and Prevention Group, Center for Public Health Sciences, National Cancer Center, Tokyo, Japan; 3 Department of Food and Life Sciences, Faculty of Food and Nutritional Sciences, Toyo University, Gunma, Japan; 4 Department of Epidemiology and Prevention, International Clinical Research Center, National Center for Global Health and Medicine, Tokyo, Japan; 5 Department of Endocrinology and Diabetes, Saitama Medical University, Saitama, Japan

**Keywords:** cohort study, Okinawan vegetable, type 2 diabetes

## Abstract

**Background:**

Some Okinawan vegetables have been reported to have anti-diabetic activity; however, no prospective cohort study has clarified whether consumption of Okinawan vegetables is associated with a risk of type 2 diabetes. This study aimed to determine the association between consumption of Okinawan vegetables and risk of type 2 diabetes through a large-scale, population-based, prospective study in Japan.

**Methods:**

We examined 10,732 participants (4,714 men and 6,018 women) aged 45–74 years who resided in Okinawa. Participants were asked to answer a 147-item food frequency questionnaire. We calculated the overall amount of Okinawan vegetables consumed and the amount of seven specific kinds of Okinawan vegetables consumed. The odds ratios (ORs) for self-reported type 2 diabetes during 5 years of follow-up were estimated via multivariate logistic regression analysis.

**Results:**

During the 5-year period, 216 new cases (123 men and 93 women) of type 2 diabetes were reported. Comparing the highest tertile to the lowest tertile of intake, the overall amount of Okinawan vegetables consumed was not associated with risk of type 2 diabetes in men (OR 1.22; 95% confidence interval [CI], 0.74–2.01, *P*-trend = 0.53) or in women (OR 0.96; 95% CI, 0.57–1.62, *P*-trend = 0.89). The consumption of seven specific kinds of Okinawan vegetables was also not associated with the risk of type 2 diabetes.

**Conclusions:**

The consumption of total Okinawan vegetables was not associated with the risk of type 2 diabetes.

## INTRODUCTION

Diabetes mellitus is a serious health condition that is becoming an increasingly important public health concern worldwide. In the Japanese population, approximately 20 million people were estimated as the sum of those strongly suspected and those with possibilities of having diabetes in 2016.^1^ The progression of diabetes can lead to complications, such as retinopathy, nephropathy, and neuropathy. These complications not only markedly lower the patient’s quality of life, but also incur high healthcare costs.^2^ Therefore, preventing type 2 diabetes is clearly an important public health priority.

Food ingredients, such as dietary fiber and magnesium, play an important role in the prevention and development of type 2 diabetes.^3^ In particular, vegetables contain high amounts of anti-oxidants, such as polyphenols, carotenoids, and ascorbic acid, and vegetable consumptions has been associated with a decreased risk of type 2 diabetes.^4^ While meta-analyses of prospective studies showed no association between vegetable and fruit intake and the risk of type 2 diabetes,^5^^–^^7^ green leafy vegetables reduced the risk of type 2 diabetes.^6^^,^^7^ Therefore, vegetables in general may not be associated with the risk of type 2 diabetes, but specific groups of vegetables may reduce its risk.

Historically, the average life expectancy of Okinawan was longer than the national average in Japan,^8^ but it is now shorter than the national average. In this study, we analyze the data from a period when longevity was high in Okinawan and assess whether the Okinawan diet may reduce the risk of type 2 diabetes. This research may contribute to improving Okinawans’ present health by assessing the benefits of the historical diet. Vegetables such as pak choi (Chingentsuai), leaf mustard, bitter gourd, Swiss chard, loofah, mugwort, and papaya are consumed more frequently in Okinawa than in other areas of Japan.^9^ These Okinawan vegetables contain high amounts of anti-oxidants, such as polyphenols, carotenoids, and ascorbic acid,^10^ and some Okinawan vegetables have been reported to exhibit anti-diabetic activity.^11^^,^^12^ These studies suggest that the consumption of Okinawan vegetables may reduce the risk of type 2 diabetes. However, to our knowledge, no prospective study has investigated the association of Okinawan vegetable intake with the risk of type 2 diabetes among the Japanese. Here, we aimed to determine the association between Okinawan vegetable intake and the risk of type 2 diabetes in the Okinawa population through a large-scale, population-based prospective cohort study. Moreover, although Kurotani et al reported that vegetable and fruit consumption was not associated with risk of type 2 diabetes in research using the Japan Public Health Center-Based Prospective Study (JPHC Study) cohort,^13^ no study has examined this topic specifically in the Okinawan population. Therefore, for comparison with consumption of Okinawan vegetables, we also examined the risk of type 2 diabetes with respect to overall vegetable and fruit consumption.

## MATERIALS AND METHODS

### Study population

The JPHC Study contained two cohorts based on the public health center (PHC) area. Cohort I started in 1990 and consisted of participants 40–59 years of age from five PHC areas. Cohort II started in 1993 and consisted of participants 40–69 years of age from six PHC areas.^14^ The JPHC Study protocol has been described previously.^15^ The study protocol and data analysis plan were approved by the institutional review board of the National Cancer Center, Tokyo, Japan, and Azabu University, respectively.

**Figure 1.**
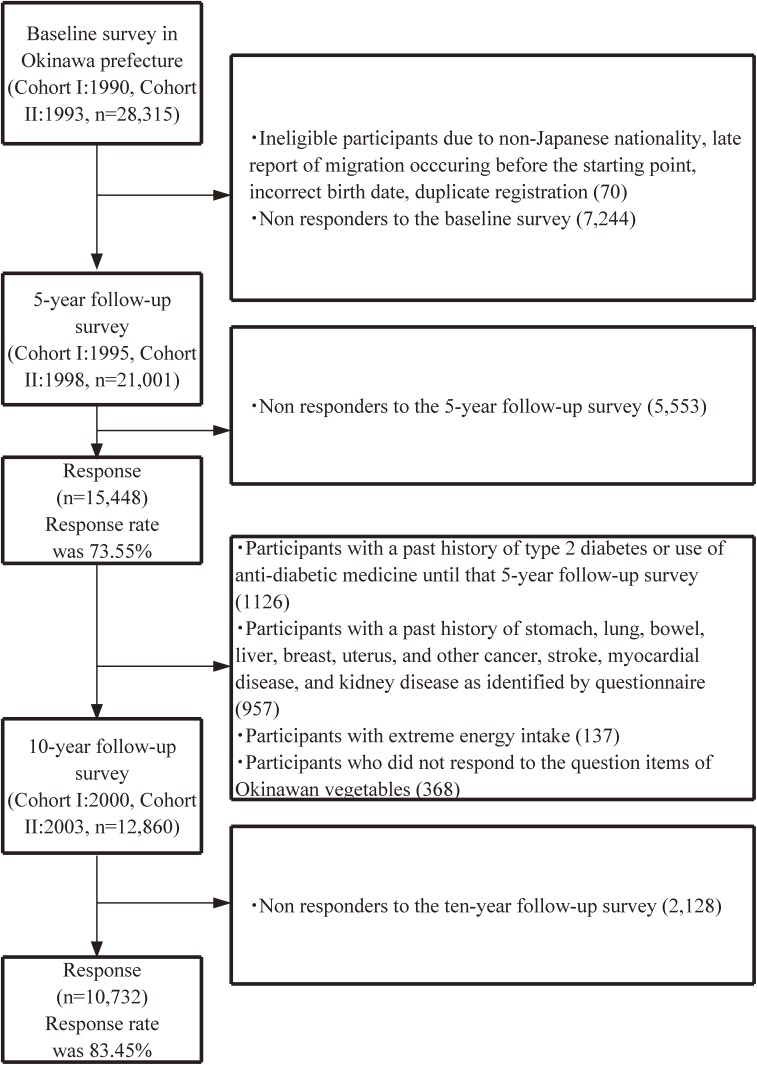
Flowchart of study participants in the Japan Public Health Center-Based Prospective Study on the association between consumption of Okinawan vegetables and risk of type 2 diabetes.

To identify the associations between Okinawan vegetable consumption and the risk of type 2 diabetes, we limited the research area to Okinawa Prefecture because the level of Okinawan vegetable intake was low in other areas. The median value of Okinawan vegetable intake was 43.7 g/day (25th–75th percentile, 22.1–78.3 g/day) in the Okinawa Prefecture, and 2.4 g/day (25th–75th percentile, 0.0–7.8 g/day) in other PHC areas. Participants consisted of 28,315 residents (14,390 men and 13,925 women) (Figure 1). At baseline survey, a self-administered questionnaire was distributed to all Cohorts I and II inhabitants in surveyed areas. The questionnaire contained a simplified version for dietary habits, as well as smoking, drinking, medical history, and demographic characteristics. For Cohort I in 1995 and Cohort II in 1998, a 5-year follow-up survey was performed using a food frequency questionnaire (FFQ). Because a FFQ contains more comprehensive information on the frequency of food intake and demographic characteristics, we used the FFQ as a starting point for assessing dietary intake. Furthermore, a 10-year follow-up survey, including a history of diabetes and use of anti-diabetic medicine, was conducted for Cohorts I and Cohort II in 2000 and 2003, respectively.

We excluded participants with duplicate registration, incorrect birth data, non-Japanese nationality, and inaccurate or late report of migration that occurred before the starting point (70 subjects), and those who were lost to follow-up before the starting point (7,244 subjects), leaving 21,001 participants. Then, we excluded those who did not respond to the 5-year follow-up survey (5,553), leaving 15,448 participants (73.6%). We further excluded participants who reported a history of diabetes, use of anti-diabetic medication (1,126 subjects), or severe disease (cancer, cerebrovascular disease, myocardial infarction, chronic liver disease, and renal disease; 957 subjects) at the 5-year follow-up survey. We also excluded participants who reported extreme total energy intake (ie, outside the mean [3 SD]; 137 subjects) and those who did not respond to the questionnaire items on Okinawan vegetables (368 subjects) at the 5-year follow-up survey. Finally, we excluded participants who did not respond to the subsequent 10-year follow-up questionnaire (2,128 subjects). A total of 10,732 participants (4,714 men and 6,018 women, 83.5%) were included in our study.

### Assessment of food consumption and nutrients

The FFQ contains 138 food items, including seven Okinawan vegetables, which contributed greatly to nutrient intake by the Okinawa participants.^16^ The FFQ used in the 5-year follow-up survey of the JPHC study has been detailed previously.^17^ Briefly, the FFQ assessed the daily intake of seven Okinawan vegetables (ie, pak choi, leaf mustard, bitter gourd, Swiss chard, loofah, mugwort, and papaya), 15 fruits, and 24 other vegetables commonly consumed in the past year.^17^ In Okinawa Prefecture, green papaya is eaten raw as salad, simmered, or stir-fried.^18^ Therefore, we included papaya as an Okinawan vegetable in our research. The daily consumption of Okinawan vegetables was calculated by multiplying the daily intake frequency by the typical portion size.

The validity and reproducibility of the FFQ were investigated in a subsample of participants in the Chubu PHC area from Cohort I and Miyako PHC area from Cohort II (56 men and 58 women for validity, 55 men and 56 women for reproducibility). The participants completed both a dietary record for a total of 28-days (Miyako PHC area) or 14-days (Chubu PHC area) and a FFQ at a 1-year interval. For Okinawan vegetables, the energy-adjusted deattenuated correlation coefficients for the validity of the FFQ in men and women were 0.17 and 0.19 and the energy-adjusted correlation coefficients for the reproducibility of the FFQ in men and women were 0.43 and 0.52, respectively. For seven specific Okinawan vegetables, the energy-adjusted deattenuated correlation coefficients for the validity of the FFQ in men and women were 0.02 and 0.60 for pak choi, −0.11 and 0.20 for leaf mustard, 0.18 and 0.20 for bitter gourd, 0.61 and 0.40 for swiss chard, 0.39 and 0.34 for loofah, 0.51 and 0.20 for mugwort, and 0.26 and 0.45 for papaya, respectively. For the reproducibility of the FFQ, energy-adjusted correlation coefficients in men and women were 0.36 and 0.54 for pak choi, 0.66 and 0.69 for leaf mustard, 0.36 and 0.46 for bitter gourd, 0.45 and 0.46 for swiss chard, 0.52 and 0.47 for loofah, 0.52 and 0.39 for mugwort, and 0.43 and 0.39 for papaya, respectively.

### Ascertainment of type 2 diabetes

Type 2 diabetes was confirmed via self-administered questionnaire. Only subjects who were newly diagnosed at the 10-year follow-up survey were considered as incident cases. Because the 5-year follow-up survey was used as the baseline survey in this study, only those who were subsequently diagnosed were regarded as incident cases during the follow-up. Kato et al reported about the validity of self-reported diabetes, 94% of cases of self-reported diabetes were confirmed in the medical records.^19^

### Statistical analysis

Analyses were performed by gender. Participants in each group were divided into tertiles of food intake (T1–T3) according to each consumer, with the lowest category as the reference. Because there were more than 50% of participants whose Swiss chard intake level was 0 g, participants were divided into two groups (0 g or >0 g) according to the amount of Swiss chard intake. To evaluate the association between diabetes risk and intake of Okinawan vegetables, specific Okinawan vegetable, multivariate logistic regression was used to estimate odds ratios (ORs), and 95% confidence intervals (CIs) were estimated using the Wald method. The first model was adjusted for age (years) and study area (two areas). In a second model assessing diabetes risk and consumption of either total Okinawan vegetables or seven specific Okinawan vegetables, we adjusted for body mass index (BMI, <21, 21–22.9, 23–24.9, 25–26.9, or ≥27 kg/m^2^); smoking status (lifetime non-smoker, former smoker, or current smoker with a consumption of either <20 or ≥20 cigarettes/day); alcohol consumption (nondrinker, occasional drinker, or drinker with a consumption of <150, 150–299, 300–449, or ≥450 g ethanol/week); metabolic equivalent task-hours per day (tertiles); family history of diabetes mellitus (yes or no); history of hypertension (yes or no); total energy intake (kcal/day, continuous); coffee consumption (g/day, continuous); and energy-adjusted dietary consumption of foods (g/d, continuous) such as meat, rice, vegetables (except for Okinawan vegetables), and fruits (except for papaya). Trend associations were assessed by assigning the ordinal numbers 0–2 to the three categories of total Okinawan vegetable and specific Okinawan vegetables consumption. An indicator variable for missing data was created for each covariate. Trend associations between confounding factors, and Okinawan vegetable intake were assessed using the Mantel-Haenszel χ^2^ test for categorical variables and linear regression analysis for continuous variables. Each food group consumption was adjusted according to the total energy intake using the residual method. In addition, to eliminate the influence of known predictors for the risk of diabetes, such as age,^20^ obesity,^21^ smoking,^22^ alcohol consumption,^23^ and menopause status,^24^ we further performed stratified analyses according to age (<65 or ≥65 years), BMI (<25 or ≥25 kg/m^2^), cigarette smoking status (smoker or non-smoker), alcohol intake (<150 or ≥150 g ethanol/week), fat intake (<median or ≥median), pork intake (<median or ≥median), red meat intake (<median or ≥median), processed meat intake (<median or ≥median), and menopause status (premenopause or postmenopause). We chose not to adjust for multiple comparisons because such adjustments could lead to possibly important findings being missed.^25^ Two-sided *P*-values <0.05 or 95% confidence intervals that did not include unity were considered statistically significant. All analyses were performed using SAS software (version 9.4; SAS Institute, Cary, NC, USA).

## RESULTS

At the 5-year follow-up survey, participants with higher intakes of Okinawan vegetables were more likely to be old. Both men and women in highest intake group of total Okinawan vegetables, had higher intakes of vegetables (except for Okinawan vegetables), fruits (except for papaya), rice, fish, coffee, and each Okinawan vegetable. They also had higher intake of protein, calcium, magnesium, dietary fiber, vitamin D, and polyunsaturated fatty acids. Only men with higher intakes of Okinawan vegetables were less likely to be an alcohol drinker. The women with higher intakes of Okinawan vegetables were more likely to be physically active and less likely to consume meat (Table 1). The median value for the consumption rate of Okinawan vegetables in total vegetable consumption was 14.5% (25th–75th percentile, 8.9–21.8%) in men, and 14.1% (25th–75th percentile, 8.7–20.7%) in women.

**Table 1.** Baseline characteristics of participants according to tertile of total Okinawan vegetables intake

	Tertiles of total Okinawan vegetables intake						*P* values^a^
	Lowest (T1)		Second (T2)		Highest (T3)		
Men							
*n*	1,560		1,561		1,560		
Age, years	57.4	(7.9)	58.2	(7.9)	58.8	(7.7)	<0.0001
BMI, kg/m^2^	24.7	(3.1)	24.5	(2.9)	24.7	(3.1)	0.9898
Total physical activity, METs	33.6	(6.6)	34.2	(6.6)	34.6	(6.7)	0.0004
Current smoker, %	34.3		33.2		29.3		0.0029
Current drinker, %	75.3		72.9		68.7		<0.0001
Family history of diabetes, %	4.9		5.6		4.4		0.5111
History of hypertension, %	14.3		14.6		15.1		0.5108
Study area (Chubu)	922	(59.1)	841	(53.9)	750	(48.1)	<0.0001
(Miyako)	638	(40.9)	720	(46.1)	810	(51.9)	
Daily food and nutrient intake							
Total energy intake, kcal	2,136	(863)	2,132	(771)	2,107	(767)	0.6572
Carbohydrate, % of energy	49.0	(12.0)	48.5	(10.4)	49.6	(9.7)	0.1515
Fat, % of energy	24.8	(8.9)	27.2	(8.2)	28.4	(7.5)	<0.0001
Protein, % of energy	12.4	(2.8)	13.2	(2.5)	13.6	(2.4)	<0.0001
Calcium, mg	422.5	(243.9)	457.9	(215.7)	522.5	(218.4)	<0.0001
Magnesium, mg	270.3	(74.0)	301.8	(80.3)	341.9	(89.2)	<0.0001
Dietary fiber, g	8.3	(3.2)	10.7	(3.4)	14.1	(4.7)	<0.0001
Vitamin D, µg	5.8	(4.1)	6.7	(3.9)	6.9	(4.3)	<0.0001
Polyunsaturated fatty acids, g	11.8	(3.6)	13.8	(3.6)	15.7	(3.7)	<0.0001
Vegetables (except for Okinawan vegetables), g	121.9	(85.7)	170.3	(93.2)	226.2	(125.4)	<0.0001
Fruits (except for papaya), g	101.0	(102.5)	126.6	(104.8)	154.3	(112.8)	<0.0001
Meat, g	86.6	(54.6)	90.2	(53.1)	83.8	(49.1)	0.2878
Rice, g	548.2	(171.7)	532.9	(149.9)	523.5	(145.1)	<0.0001
Fish, g	59.5	(47.3)	70.1	(42.4)	72.4	(43.7)	<0.0001
Coffee, g	217.8	(288.0)	179.7	(214.6)	160.1	(199.6)	<0.0001
Total intake of Okinawan vegetables, g	17.2	(7.2)	44.5	(9.8)	113.7	(65.7)	<0.0001
Pak choi	1.6	(2.5)	4.6	(5.7)	10.9	(15.9)	<0.0001
Leaf mustard	1.7	(2.2)	4.5	(5.1)	11.7	(15.0)	<0.0001
Bitter gourd	7.0	(5.2)	18.6	(10.8)	45.3	(38.4)	<0.0001
Swiss chard	0.9	(2.1)	2.5	(4.5)	8.6	(18.0)	<0.0001
Loofah	3.1	(3.0)	8.4	(7.3)	25.3	(25.7)	<0.0001
Mugwort	0.2	(0.5)	0.3	(0.5)	0.5	(0.9)	<0.0001
Papaya	2.5	(2.7)	5.2	(5.9)	9.9	(13.9)	<0.0001

Women							
*n*	1,997		1,998		1,998		
Age, years	57.9	(8.2)	58.0	(7.9)	58.9	(8.0)	0.0009
BMI, kg/m^2^	24.3	(3.3)	24.3	(3.3)	24.5	(3.2)	0.0682
Total physical activity, METs	32.6	(5.7)	33.3	(5.7)	33.4	(5.9)	<0.0001
Current smoker, %	3.5		2.8		2.1		0.0124
Current drinker, %	9.8		9.1		7.0		0.0016
Family history of diabetes, %	6.0		5.0		6.5		0.5453
History of hypertension, %	12.8		11.6		14.8		0.0611
Study area (Chubu)	1,211	(60.0)	1,127	(56.4)	1,040	(52.1)	<0.0001
(Miyako)	786	(39.4)	871	(43.6)	958	(47.9)	
Daily food and nutrient intake							
Total energy intake, kcal	1,811	(745)	1,813	(662)	1,776	(638)	0.3787
Carbohydrate, % of energy	52.4	(10.6)	52.4	(9.2)	53.3	(8.3)	0.0174
Fat, % of energy	31.0	(9.2)	31.4	(7.8)	31.2	(7.0)	0.2543
Protein, % of energy	14.2	(2.4)	14.5	(2.3)	14.5	(2.2)	0.0001
Calcium, mg	474.9	(252.3)	501.6	(217.8)	558.7	(207.5)	<0.0001
Magnesium, mg	265.7	(74.6)	292.2	(73.2)	334.3	(82.2)	<0.0001
Dietary fiber, g	9.7	(3.6)	11.8	(3.5)	15.3	(4.6)	<0.0001
Vitamin D, µg	5.7	(3.4)	6.3	(4.3)	6.4	(4.1)	<0.0001
Polyunsaturated fatty acids, g	12.7	(3.3)	13.8	(3.1)	15.3	(3.3)	<0.0001
Vegetables (except for Okinawan vegetables), g	149.7	(106.8)	186.6	(108.2)	242.7	(121.2)	<0.0001
Fruits (except for papaya), g	129.7	(122.3)	157.8	(120.3)	176.8	(121.8)	<0.0001
Meat, g	83.2	(51.1)	77.1	(44.8)	65.3	(40.8)	<0.0001
Rice, g	474.7	(135.0)	455.4	(117.0)	444.4	(118.0)	<0.0001
Fish, g	55.6	(40.8)	62.9	(41.1)	64.5	(40.5)	<0.0001
Coffee, g	151.7	(178.6)	150.5	(167.4)	128.8	(154.6)	<0.0001
Total intake of Okinawan vegetables, g	20.4	(8.2)	50.0	(10.1)	123.5	(75.4)	<0.0001
Pak choi	2.3	(3.3)	5.9	(6.9)	13.6	(16.0)	<0.0001
Leaf mustard	2.1	(2.8)	5.1	(5.5)	12.7	(16.1)	<0.0001
Bitter gourd	7.8	(5.7)	19.7	(11.2)	45.5	(37.1)	<0.0001
Swiss chard	1.0	(2.2)	3.0	(5.4)	9.8	(20.7)	<0.0001
Loofah	3.6	(3.5)	9.6	(7.8)	26.7	(29.4)	<0.0001
Mugwort	0.2	(0.5)	3.0	(5.4)	0.5	(1.0)	<0.0001
Papaya	3.2	(3.3)	6.0	(6.8)	13.0	(24.6)	<0.0001

Values are expressed as mean (standard deviation) or number (%).

^a^Determined by using Chi-square test for categorical variables and linear regression analysis for continuous variables with assignment of ordinal numbers 0–3 to categories of total Okinawan vegetable intake.

During the 5-year period, 216 new cases (123 men and 93 women) of type 2 diabetes were self-reported. There was no statistically significant association with the risk of type 2 diabetes and intakes of Okinawan vegetables. Comparing the highest tertile to the lowest tertile of intake, the overall amount of Okinawan vegetables consumed was not associated with risk of type 2 diabetes in men (OR 1.22; 95% confidence interval [CI], 0.74–2.01, *P*-trend = 0.53) or in women (OR 0.96; 95% CI, 0.57–1.62, *P*-trend = 0.89). The seven specific Okinawan vegetables were not associated with the risk of type 2 diabetes in both men and women (Table 2 and Table 3). In Table 2 and Table 3, we also analyzed without adjusting the history of hypertension. No significant associations were found (data not shown). We also observed no statistically significant association between the risk of type 2 diabetes and intake of vegetables, fruits, or combined vegetables and fruits in both men and women (eTable 1). In addition, stratified analyses according to menopausal status also showed no association (eTable 2). Further stratified analyses according to age, BMI, cigarette smoking status, alcohol intake, fat intake, pork intake, red meat intake, and processed meat intake also showed no meaningful associations (data not shown).

**Table 2.** Odds ratios and 95% confidence intervals of type 2 diabetes in a cohort of 4,714 men according to tertiles of total or specific Okinawan vegetables, total vegetables, total fruits, or total vegetables and fruits combined

Men	T1	T2		T3		*P* for trend
		
	Reference	OR	95% CI	OR	95% CI	
			lower–upper		lower–upper	
Total Okinawan vegetables						
Median intake, g/day	16.7	44.1		113.2		
Number of cases/participants	35/1,571	47/1,572		41/1,571		
Age- and area-adjusted^a^	1.0	1.35	(0.86–2.10)	1.17	(0.74–1.85)	0.52
Multivariate-adjusted^b^	1.0	1.38	(0.88–2.19)	1.22	(0.74–2.01)	0.53

Pak choi						
Median intake, g/day	0.0	2.4		15.0		
Number of cases/participants	37/1,648	47/1,533		39/1,533		
Age- and area-adjusted^a^	1.0	1.38	(0.89–2.15)	1.13	(0.71–1.80)	0.62
Multivariate-adjusted^b^	1.0	1.42	(0.90–2.22)	1.15	(0.71–1.86)	0.64

Leaf mustard						
Median intake, g/day	0.2	2.6		15.0		
Number of cases/participants	42/1,571	51/1,572		30/1,571		
Age- and area-adjusted^a^	1.0	1.18	(0.77–1.80)	0.67	(0.40–1.09)	0.13
Multivariate-adjusted^b^	1.0	1.19	(0.77–1.83)	0.68	(0.41–1.13)	0.15

Bitter gourd						
Median intake, g/day	4.8	16.6		49.0		
Number of cases/participants	42/1,571	34/1,572		47/1,571		
Age- and area-adjusted^a^	1.0	0.80	(0.51–1.27)	1.12	(0.74–1.71)	0.57
Multivariate-adjusted^b^	1.0	0.80	(0.50–1.27)	1.15	(0.74–1.80)	0.60

Swiss chard						
Median intake, g/day	0.0	10.0				
Number of cases/participants	77/2,846	46/1,868				
Age- and area-adjusted^a^	1.0	0.91	(0.63–1.32)			0.63
Multivariate-adjusted^b^	1.0	0.91	(0.62–1.33)			0.61

Loofah						
Median intake, g/day	1.4	6.2		28.8		
Number of cases/participants	45/1,571	36/1,572		42/1,571		
Age- and area-adjusted^a^	1.0	0.79	(0.51–1.23)	0.91	(0.59–1.41)	0.69
Multivariate-adjusted^b^	1.0	0.78	(0.49–1.22)	0.90	(0.57–1.41)	0.61

Mugwort						
Median intake, g/day	0.0	0.2		0.8		
Number of cases/participants	37/1,571	35/1,572		51/1,571		
Age- and area-adjusted^a^	1.0	0.95	(0.60–1.52)	1.40	(0.91–2.16)	0.11
Multivariate-adjusted^b^	1.0	0.99	(0.61–1.59)	1.39	(0.89–2.17)	0.16

Papaya						
Median intake, g/day	0.7	3.2		13.5		
Number of cases/participants	38/1,571	37/1,572		48/1,571		
Age- and area-adjusted^a^	1.0	0.98	(0.62–1.55)	1.29	(0.83–1.99)	0.24
Multivariate-adjusted^b^	1.0	0.99	(0.63–1.58)	1.33	(0.85–2.08)	0.23

^a^Adjusted for age (years) and study area (two areas).

^b^Adjusted for age (years) and study area (two areas), bmi (kg/m^2^; <21, 21–22.9, 23–24.9, 25–26.9, or ≧27), cigarette smoking status, alcohol consumption (g ethanol/wk; <150, 150–299, 300–449, or ≧450), METs (tertile), history of hypertension, family history of diabetes, energy intake, coffee consumption, intakes of meat (g/d), rice (g/d), vegetables (g/d, except for Okinawan vegetables), fruits (g/d, except for papaya).

**Table 3.** Odds ratios and 95% confidence intervals of type 2 diabetes in a cohort of 6,018 women according to tertiles of total or specific Okinawan vegetables, total vegetables, total fruits, or total vegetables and fruits combined

Women	T1	T2		T3		*P* for trend
		
	Reference	OR	95% CI	OR	95% CI	
			lower–upper		lower–upper	
Total Okinawan vegetables						
Median intake, g/day	20.0	49.7		123.1		
Number of cases/participants	34/2,006	25/2,006		34/2,006		
Age- and area-adjusted^a^	1.0	0.73	(0.43–1.23)	0.98	(0.60–1.58)	0.93
Multivariate-adjusted^b^	1.0	0.78	(0.46–1.33)	0.96	(0.57–1.62)	0.89

Pak choi						
Median intake, g/day	0.2	3.4		18.2		
Number of cases/participants	39/2,006	23/2,006		31/2,006		
Age- and area-adjusted^a^	1.0	0.62	(0.36–1.04)	0.83	(0.51–1.36)	0.42
Multivariate-adjusted^b^	1.0	0.65	(0.38–1.11)	0.88	(0.53–1.46)	0.53

Leaf mustard						
Median intake, g/day	0.2	3.0		16.4		
Number of cases/participants	30/2,006	30/2,006		33/2,006		
Age- and area-adjusted^a^	1.0	1.06	(0.63–1.79)	1.19	(0.70–2.01)	0.51
Multivariate-adjusted^b^	1.0	1.04	(0.61–1.77)	1.31	(0.76–2.26)	0.34

Bitter gourd						
Median intake, g/day	5.2	17.5		49.8		
Number of cases/participants	36/2,006	27/2,006		30/2,006		
Age- and area-adjusted^a^	1.0	0.74	(0.44–1.21)	0.80	(0.49–1.32)	0.38
Multivariate-adjusted^b^	1.0	0.75	(0.45–1.26)	0.81	(0.48–1.36)	0.40

Swiss chard						
Median intake, g/day	0.0	10.7				
Number of cases/participants	48/3,433	45/2,585				
Age- and area-adjusted^a^	1.0	1.23	(0.82–1.86)			0.32
Multivariate-adjusted^b^	1.0	1.29	(0.85–1.97)			0.25

Loofah						
Median intake, g/day	1.6	7.2		30.8		
Number of cases/participants	26/2,006	37/2,006		30/2,006		
Age- and area-adjusted^a^	1.0	1.44	(0.87–2.40)	1.15	(0.67–1.97)	0.62
Multivariate-adjusted^b^	1.0	1.36	(0.81–2.28)	1.14	(0.65–1.98)	0.68

Mugwort						
Median intake, g/day	0.0	0.2		0.9		
Number of cases/participants	30/2,006	26/2,006		37/2,006		
Age- and area-adjusted^a^	1.0	0.86	(0.51–1.46)	1.19	(0.73–1.94)	0.46
Multivariate-adjusted^b^	1.0	0.88	(0.52–1.51)	1.10	(0.66–1.82)	0.75

Papaya						
Median intake, g/day	1.1	3.6		17.4		
Number of cases/participants	29/2,006	31/2,006		33/2,006		
Age- and area-adjusted^a^	1.0	1.06	(0.64–1.77)	1.11	(0.67–1.85)	0.68
Multivariate-adjusted^b^	1.0	1.08	(0.65–1.82)	1.24	(0.73–2.08)	0.48

^a^Adjusted for age (years) and study area (two areas).

^b^Adjusted for age (years) and study area (two areas), bmi (kg/m^2^; <21, 21–22.9, 23–24.9, 25–26.9, or ≧27), cigarette smoking status, alcohol consumption (g ethanol/wk; <150, 150–299, 300–449, or ≧450), METs (tertile), history of hypertension, family history of diabetes, energy intake, coffee consumption, intakes of meat (g/d), rice (g/d), vegetables (g/d, except for Okinawan vegetables), fruits (g/d, except for papaya).

## DISCUSSION

In this large population-based prospective study of Japanese adults, the consumption of Okinawan vegetables overall and of seven specific Okinawan vegetables was not found to be statistically associated with the risk of type 2 diabetes in either men or women. Additionally, we found no significant association between the intake of vegetables, fruits, and vegetables and fruits combined and the risk of type 2 diabetes. To our knowledge, this was the first study to examine the association of Okinawan vegetable intake with type 2 diabetes.

The current findings revealed the null association between intake of total Okinawan vegetables, vegetables, fruits, and vegetables and fruits combined and the risk of type 2 diabetes in the Japanese population. Vegetables and fruit are rich in dietary fiber and magnesium, which have been reported to improve insulin resistance.^26^^,^^27^ However, a meta-analysis of prospective studies showed no association between vegetable and fruit fiber and the risk of type 2 diabetes.^7^ In addition, our previous reports showed that vegetable and fruit intake^13^ or magnesium intake^28^ was not associated with the risk of type 2 diabetes in the Japanese population. Thus, the results of this study are consistent with those of previous studies. In contrast, previous research reported that intakes of cereal fiber were inversely associated with the risk of type 2 diabetes.^7^ The protective effect of cereal fiber on type 2 diabetes may exerted through the modulation of gut microbiota.^29^ Therefore, the effect of dietary fiber on the risk of type 2 diabetes may vary depending on the types of food.

Oxidative stress plays a major role in insulin resistance due to insulin signal disruption.^30^ Previous studies either showed a negative association or no association between risk of type 2 diabetes and vitamins E, C, carotenoid, and lycopene.^5^^,^^31^^–^^35^ A meta-analysis of prospective studies showed that the intake of antioxidants was associated with a 13% reduction in the risk of type 2 diabetes, which was mainly attributed to the intake of vitamin E and carotenoids.^5^ However, our result showed that the seven specific Okinawan vegetables that contained high amounts of anti-oxidants, such as carotenoids,^10^^,^^36^ were not associated with the risk of type 2 diabetes (Table 2 and Table 3). In addition, we investigated the association between dietary intakes of three specific carotenoids (α-carotene, β-carotene, and cryptoxanthin) and total carotenoid (sum of three specific carotenoids); no association was observed (data not shown).

Previous studies indicate an apparent association between sex and oxidative stress. A study showed that in vivo biomarkers of oxidative stress were higher in young men than in women of the same age.^37^ Estradiol exerts anti-oxidative activity,^38^ and postmenopausal status increases the risk of type 2 diabetes.^24^ Therefore, men may benefit more from dietary anitioxidants than women; women may be more susceptible after menopause. In our study, there was no association between Okinawan vegetable intake and risk of type 2 diabetes in both men and women (Table 2 and Table 3), and no association was noted in the stratified analyses according to menopausal status (premenopausal or postmenopausal, eTable 2).

Kaempferol, a dietary flavonoid present in leaf mustard, exerts an anti-oxidant activity^39^ and improves hyperglycemia by ameliorating insulin resistance in diet-induced obese mice.^11^ The present study showed a marginally protective association of leaf mustard intake with the risk of type 2 diabetes only in men (Table 2). However, the validity of the FFQ for leaf mustard intake is remarkably low. This could be attributed solely to chance, and further investigation is necessary.

Okinawan vegetables are commonly eaten by stir-frying and are often eaten with processed meat or red meat, such as pork. Several cohort studies have shown no association between total fat intake and risk of type 2 diabetes.^40^^,^^41^ However, some studies have reported a positive association.^42^^,^^43^ A meta-analysis of prospective studies showed that intake of red and processed meat was positively and significantly associated with risk of type 2 diabetes.^44^ Therefore, the association between Okinawan vegetables and the risk of type 2 diabetes may be attenuated by the influence of fat, red meat, and processed meat. We further analyzed this association according to fat, pork, red meat, and processed meat intake. We found no meaningful results (data not shown). Further stratified analyses according to age, BMI, cigarette smoking status, and alcohol intake also showed no meaningful associations (data not shown). Our previous research using all area of JPHC study have reported that green leafy vegetable intake reduced the risk of type 2 diabetes in Japanese population.^13^ Therefore, we also analyzed associations between Okinawan green leafy vegetable (pak choi, leaf mustard, swiss chard, and mugwort) intake and the risk of type 2 diabetes; no association was observed (data not shown). In the present study, the range of Okinawan vegetable and seven specific Okinawan vegetable intakes were narrow; therefore, the variation was small. This may have attenuated the association between intake of Okinawan vegetables overall or of the seven specific Okinawan vegetables and the risk of type 2 diabetes.

The strengths of this study include the population-based prospective design based on a large number of participants both men and women, and adjustment or stratification with potentially important confounding variables. However, there are some limitations to note. First, the FFQ had low validity for estimating the intake of Okinawan vegetables overall and of the even specific Okinawan vegetables due to the small variation from the narrow range of intake. Thus, the association between Okinawan vegetable intake and the risk of type 2 diabetes may have been attenuated. However, at present, Okinawan vegetables are widely distributed not only in Okinawa Prefecture but throughout Japan, and thus the number of people consuming Okinawan vegetables throughout Japan constitute a larger sample and larger variation than in this research. Therefore, further investigation is required to verify the association between Okinawan vegetable intake and the risk of type 2 diabetes. Second, the diagnosis of type 2 diabetes was ascertained via self-reports, the incidence of diabetes in our study may be lower than that defined using laboratory data. A validation study conducted in three of JPHC cohort areas including Okinawa area, 94% of self-reported diabetes cases were confirmed to have diabetes after a medical chart review.^19^ Nevertheless, the misclassification of diagnosis of type 2 diabetes may have distorted the results to some extent. Third, dietary assessments were only performed once. Therefore, the changes of dietary pattern not be reflected, and there is a possibility that underestimation via misclassification may have occurred on dietary intake. Fourth, the follow-up period was relatively short (5 years). To evaluate the preventive effect of Okinawan vegetables through improvement of insulin resistance, longer follow-up period may be required. Finally, we could not rule out the possibility of unmeasured and residual confounding.

In conclusion, this prospective study shows that the consumption of total Okinawan vegetables was not associated with the risk of type 2 diabetes among the residents of Okinawan Prefecture. Further studies on the relationship between specific Okinawan vegetables and the risk of type 2 diabetes are required.

## APPENDIX A. SUPPLEMENTARY DATA

eTable 1. Odds ratios and 95% confidence intervals of type 2 diabetes in a cohort of 4714 men and 6018 women according to tertiles of total vegetables, total fruits, or total vegetables and fruits combined

eTable 2. Odds ratios and 95% confidence intervals of type 2 diabetes according to tertiles of Okinawan vegetables: stratified analyses according to the menopausal status
