# Supplementary material for: Association Between Okinawan Vegetables Consumption and Risk of Type 2 Diabetes in Japanese Communities: The JPHC Study
Source: J Epidemiol. 2020 May 5;30(5):227–35. doi: 10.2188/jea.JE20180262 (PMC7153960; doi:10.2188/jea.JE20180262)
Supplement: Supplementary file 1 [file je-30-227-s001.pdf]

eTable 1. Odds ratios and 95% confidence intervals of type 2 diabetes in a cohort of 4714 men and 6018 women according to tertiles of total vegetables, total fruits, or total vegetables and fruits combined

		T1	T2		T3		p for trend
		Reference	OR	95% CI lower − upper	OR	95% CI lower − upper	
Men							
Total Vegetables							
	Median intake, g/day	100.7	199.6		372.2		
	Number of cases/participants	39/1571	40/1572		44/1571		
	Age- and area-adjusted[a]	1.0	1.02	(0.65 − 1.60)	1.12	(0.72 − 1.75)	0.59
	Multivariate-adjusted[b]	1.0	1.07	(0.68 − 1.70)	1.31	(0.83 − 2.10)	0.24
Total Fruits							
	Median intake, g/day	34.7	111.6		251.7		
	Number of cases/participants	41/1571	45/1572		37/1571		
	Age- and area-adjusted[a]	1.0	1.10	(0.72 − 1.69)	0.89	(0.57 − 1.41)	0.65
	Multivariate-adjusted[c]	1.0	1.15	(0.74 − 1.78)	0.94	(0.59 − 1.51)	0.72
Total vegetables and Fruits							
	Median intake, g/day	167.5	326.6		575.4		
	Number of cases/participants	39/1571	44/1572		40/1571		
	Age- and area-adjusted[a]	1.0	1.13	(0.73 − 1.75)	1.02	(0.65 − 1.60)	0.92
	Multivariate-adjusted[d]	1.0	1.16	(0.75 − 1.82)	1.19	(0.74 − 1.92)	0.47
Women							
Total Vegetables							
	Median intake, g/day	118.9	223.7		405.3		
	Number of cases/participants	29/2006	33/2006		31/2006		
	Age- and area-adjusted[a]	1.0	1.13	(0.68 − 1.87)	1.03	(0.62 − 1.72)	0.92
	Multivariate-adjusted[b]	1.0	1.21	(0.73 − 2.03)	1.12	(0.64 − 1.95)	0.73
Total Fruits							
	Median intake, g/day	52.2	137.7		294.5		
	Number of cases/participants	33/2006	32/2006		28/2006		
	Age- and area-adjusted[a]	1.0	0.98	(0.60 − 1.60)	0.86	(0.51 − 1.42)	0.55
	Multivariate-adjusted[c]	1.0	1.06	(0.64 − 1.75)	0.91	(0.53 − 1.57)	0.71
Total vegetables and Fruits							
	Median intake, g/day	209.9	379.2		640.7		
	Number of cases/participants	29/2006	29/2006		35/2006		
	Age- and area-adjusted[a]	1.0	0.99	(0.59 − 1.66)	1.18	(0.72 − 1.94)	0.51
	Multivariate-adjusted[d]	1.0	1.01	(0.64 − 1.88)	1.40	(0.80 − 2.43)	0.26

[a] Adjusted for age (y) and study area (2 areas).

[b] Adjusted for age (y) and study area (2 areas), bmi (kg/m2; <21, 21–22.9, 23–24.9, 25–26.9, or ≧27), cigarette smoking status, alcohol consumption (g ethanol/wk; <150, 150–299, 300–449, or ≧450), METs (tertile), history of hypertension, family history of diabetes, energy intake, coffee consumption, intakes of meat (g/d), rice (g/d), fruits(g/d).

[c] Adjusted for age (y) and study area (2 areas), bmi (kg/m2; <21, 21–22.9, 23–24.9, 25–26.9, or ≧27), cigarette smoking status, alcohol consumption (g ethanol/wk; <150, 150–299, 300–449, or ≧450), METs (tertile), history of hypertension, family history of diabetes, energy intake, coffee consumption, intakes of meat (g/d), rice (g/d), vegetables (g/d).

[d] Adjusted for age (y) and study area (2 areas), bmi (kg/m2; <21, 21–22.9, 23–24.9, 25–26.9, or ≧27), cigarette smoking status, alcohol consumption (g ethanol/wk; <150, 150–299, 300–449, or ≧450), METs (tertile), history of hypertension, family history of diabetes, energy intake, coffee consumption, intakes of meat (g/d), rice (g/d).

eTable 2. Odds ratios and 95% confidence intervals of type 2 diabetes according to tertiles of Okinawan vegetables: stratified analyses according to the menopausal status

Women			T1	T2		T3		
			Reference	OR	95% CI lower − upper	OR	95% CI lower − upper	p for trend
Total Okinawan vegetables								
	Premenopausal	Number of cases/participants	7/485	5/452		5/378		
		Age- and area-adjusted[a]	1.0	0.75	(0.23 − 2.37)	0.86	(0.27 − 2.73)	0.79
		Multivariate-adjusted[b]	1.0	0.80	(0.24 − 2.67)	1.11	(0.30 − 4.21)	0.85
	Postmenopausal	Number of cases/participants	25/1424	20/1459		29/1526		
		Age- and area-adjusted[a]	1.0	0.79	(0.43 − 1.42)	1.11	(0.64 − 1.90)	0.70
		Multivariate-adjusted[b]	1.0	0.83	(0.45 − 1.53)	1.06	(0.59 − 1.92)	0.86
Pak choi								
	Premenopausal	Number of cases/participants	5/339	4/496		8/480		
		Age- and area-adjusted[a]	1.0	0.50	(0.13 − 1.90)	0.99	(0.31 − 3.15)	0.97
		Multivariate-adjusted[b]	1.0	0.56	(0.14 − 2.21)	1.50	(0.42 − 5.33)	0.67
	Postmenopausal	Number of cases/participants	32/1535	19/1421		23/1453		
		Age- and area-adjusted[a]	1.0	0.65	(0.37 − 1.16)	0.79	(0.46 − 1.38)	0.38
		Multivariate-adjusted[b]	1.0	0.71	(0.40 − 1.27)	0.83	(0.47 − 1.48)	0.45
Leaf mustard								
	Premenopausal	Number of cases/participants	2/370	7/462		8/483		
		Age- and area-adjusted[a]	1.0	2.60	(0.52 − 12.99)	2.74	(0.54 − 13.83)	0.25
		Multivariate-adjusted[b]	1.0	2.64	(0.51 − 13.81)	3.61	(0.66 − 19.86)	0.19
	Postmenopausal	Number of cases/participants	27/1523	23/1455		24/1431		
		Age- and area-adjusted[a]	1.0	0.94	(0.53 − 1.66)	1.04	(0.58 − 1.87)	0.89
		Multivariate-adjusted[b]	1.0	0.96	(0.54 − 1.70)	1.17	(0.64 − 2.15)	0.64
Bitter gourd								
	Premenopausal	Number of cases/participants	8/476	4/447		5/392		
		Age- and area-adjusted[a]	1.0	0.54	(0.13 − 1.81)	0.76	(0.25 − 2.34)	0.60
		Multivariate-adjusted[b]	1.0	0.56	(0.16 − 1.95)	0.92	(0.27 − 3.16)	0.86
	Postmenopausal	Number of cases/participants	26/1415	23/1475		25/1519		
		Age- and area-adjusted[a]	1.0	0.83	(0.47 − 1.47)	0.88	(0.51 − 1.54)	0.66
		Multivariate-adjusted[b]	1.0	0.86	(0.48 − 1.53)	0.87	(0.48 − 1.56)	0.59
Swiss chard								
	Premenopausal	Number of cases/participants	6/804	11/511				
		Age- and area-adjusted[a]	1.0	1.78	(0.65 − 4.86)			0.26
		Multivariate-adjusted[b]	1.0	1.55	(0.53 − 4.55)			0.36
	Postmenopausal	Number of cases/participants	40/2450	34/1959				
		Age- and area-adjusted[a]	1.0	0.95	(0.58 − 1.55)			0.86
		Multivariate-adjusted[b]	1.0	1.07	(0.67 − 1.71)			0.79
Loofah								
	Premenopausal	Number of cases/participants	3/484	10/429		4/402		
		Age- and area-adjusted[a]	1.0	3.69	(0.98 − 13.54)	1.44	(0.32 − 6.56)	0.77
		Multivariate-adjusted[b]	1.0	3.70	(0.98 − 13.93)	1.68	(0.34 − 8.23)	0.58
	Postmenopausal	Number of cases/participants	21/1407	27/1495		26/1507		
		Age- and area-adjusted[a]	1.0	1.25	(0.70 − 2.22)	1.22	(0.68 − 2.19)	0.51
		Multivariate-adjusted[b]	1.0	1.18	(0.66 − 2.12)	1.18	(0.64 − 2.16)	0.65
Mugwort								
	Premenopausal	Number of cases/participants	8/510	4/464		5/341		
		Age- and area-adjusted[a]	1.0	0.73	(0.41 − 1.29)	0.96	(0.55 − 1.68)	0.86
		Multivariate-adjusted[b]	1.0	0.75	(0.42 − 1.33)	0.95	(0.53 − 1.69)	0.86
	Postmenopausal	Number of cases/participants	27/1382	18/1473		29/1554		
		Age- and area-adjusted[a]	1.0	0.62	(0.34 − 1.13)	0.93	(0.55 − 1.59)	0.79
		Multivariate-adjusted[b]	1.0	0.64	(0.35 − 1.18)	0.87	(0.50 − 1.50)	0.53
Papaya								
	Premenopausal	Number of cases/participants	6/454	5/470		6/391		
		Age- and area-adjusted[a]	1.0	0.81	(0.25 − 2.69)	1.26	(0.40 − 3.98)	0.69
		Multivariate-adjusted[b]	1.0	0.77	(0.22 − 2.67)	1.42	(0.43 − 4.74)	0.45
	Postmenopausal	Number of cases/participants	22/1451	25/1444		27/1514		
		Age- and area-adjusted[a]	1.0	1.12	(0.63 − 2.00)	1.12	(0.63 − 1.99)	0.49
		Multivariate-adjusted[b]	1.0	1.17	(0.65 − 2.11)	1.28	(0.71 − 2.30)	0.49
Total Vegetables								
	Premenopausal	Number of cases/participants	6/515	7/433		4/367		
		Age- and area-adjusted[a]	1.0	1.37	(0.46 − 4.10)	0.92	(0.26 − 3.28)	0.90
		Multivariate-adjusted[c]	1.0	1.47	(0.47 − 4.57)	1.11	(0.27 − 4.57)	0.68
	Postmenopausal	Number of cases/participants	22/1379	25/1489		27/1541		
		Age- and area-adjusted[a]	1.0	1.06	(0.59 − 1.88)	1.10	(0.62 − 1.93)	0.75
		Multivariate-adjusted[c]	1.0	1.13	(0.63 − 2.04)	1.17	(0.63 − 2.16)	0.80
Total Fruits								
	Premenopausal	Number of cases/participants	6/447	7/420		4/448		
		Age- and area-adjusted[a]	1.0	1.27	(0.42 − 3.81)	0.68	(0.19 − 2.44)	0.59
		Multivariate-adjusted[d]	1.0	1.67	(0.53 − 5.27)	0.91	(0.22 − 3.70)	0.97
	Postmenopausal	Number of cases/participants	26/1440	25/1500		23/1469		
		Age- and area-adjusted[a]	1.0	0.93	(0.53 − 1.62)	0.87	(0.49 − 1.52)	0.59
		Multivariate-adjusted[d]	1.0	0.99	(0.56 − 1.75)	0.90	(0.50 − 1.65)	0.68
Total vegetables and Fruits								
	Premenopausal	Number of cases/participants	5/507	6/414		6/394		
		Age- and area-adjusted[a]	1.0	1.50	(0.45 − 4.94)	1.59	(0.48 − 5.25)	0.46
		Multivariate-adjusted[e]	1.0	2.04	(0.57 − 7.23)	2.70	(0.67 − 10.91)	0.15
	Postmenopausal	Number of cases/participants	23/1390	22/1495		29/1524		
		Age- and area-adjusted[a]	1.0	0.89	(0.49 − 1.60)	1.15	(0.66 − 1.99)	0.62
		Multivariate-adjusted[e]	1.0	0.97	(0.53 − 1.79)	1.29	(0.69 − 2.40)	0.47

[a] Adjusted for age (y) and study area (2 areas).

[b] Adjusted for age (y) and study area (2 areas), bmi (kg/m2; <21, 21–22.9, 23–24.9, 25–26.9, or ≧27), cigarette smoking status, alcohol consumption (g ethanol/wk; <150, 150–299, 300–449, or ≧450), METs (tertile), history of hypertension, family history of diabetes, energy intake, coffee consumption, intakes of meat (g/d), rice (g/d), vegetables (g/d, except for Okinawan vegetables) , fruits (g/d, except for papaya)

[c] Adjusted for age (y) and study area (2 areas), bmi (kg/m2; <21, 21–22.9, 23–24.9, 25–26.9, or ≧27), cigarette smoking status, alcohol consumption (g ethanol/wk; <150, 150–299, 300–449, or ≧450), METs (tertile), history of hypertension, family history of diabetes, energy intake, coffee consumption, intakes of meat (g/d), rice (g/d), fruits(g/d).

[d] Adjusted for age (y) and study area (2 areas), bmi (kg/m2; <21, 21–22.9, 23–24.9, 25–26.9, or ≧27), cigarette smoking status, alcohol consumption (g ethanol/wk; <150, 150–299, 300–449, or ≧450), METs (tertile), history of hypertension, family history of diabetes, energy intake, coffee consumption, intakes of meat (g/d), rice (g/d), vegetables (g/d).

[e] Adjusted for age (y) and study area (2 areas), bmi (kg/m2; <21, 21–22.9, 23–24.9, 25–26.9, or ≧27), cigarette smoking status, alcohol consumption (g ethanol/wk; <150, 150–299, 300–449, or ≧450), METs (tertile), history of hypertension, family history of diabetes, energy intake, coffee consumption, intakes of meat (g/d), rice (g/d).
